# Analysis of the Psychosocial Sphere of Older Adults in Extreme Poverty in the Peruvian Amazon

**DOI:** 10.3390/healthcare11233022

**Published:** 2023-11-23

**Authors:** María Teresa Murillo-Llorente, Nerea Caballero Coloma, Francisco Tomás-Aguirre, Manuel Tejeda-Adell, Ignacio Ventura, Marcelino Perez-Bermejo

**Affiliations:** 1 SONEV Research Group, School of Medicine and Health Sciences, Catholic University of Valencia San Vicente Mártir, C/Quevedo nº 2, 46001 Valencia, Spain; mt.murillo@ucv.es; 2 School of Medicine and Health Sciences, Catholic University of Valencia San Vicente Mártir, C/Quevedo nº 2, 46001 Valencia, Spain; nerea.caballero@mail.ucv.es (N.C.C.); paco.tomas@ucv.es (F.T.-A.); manuel.tejeda@ucv.es (M.T.-A.); ignacio.ventura@ucv.es (I.V.)

**Keywords:** older adults, quality of life, extreme poverty, loneliness, family support, social situation, cognitive status, depression

## Abstract

The situation of social exclusion in which older adults live in extreme poverty is a problem that leads to psychological alterations such as depression or cognitive deterioration. Our objective was to analyze the living conditions and the psychosocial sphere of older adult people living in extreme poverty in Requena del Tapiche in Peru. This was an observational, descriptive, cross-sectional study. Sixty participants between 60 and 100 years of age of both sexes were included who gave their informed consent. Sociodemographic variables were analyzed, and the Gijón, family Apgar, Yesavage, and Pfeiffer scales were used. The sample was composed of 55% women and 45% men, with a mean age of 79.2 years (SD 6.67). More than half live alone or with their spouse. Fifty-seven percent sleep on the floor or on wood, and about 82% do not have safe water. Family dysfunction is found in 40%, and 98% are at social risk or with an established social problem and a precarious economic situation. More than 60% suffer from depressive symptoms, which are more frequent in women. We conclude that older adults perceive deficient family support, observing a deteriorated social situation. Most of them are at risk of social exclusion and loneliness, making them more vulnerable. They show sadness, with a high rate of depression. People with more cognitive impairment live alone, and those in social exclusion suffer a higher degree of depression. More cooperative projects and health promotion interventions developed in the peripheral neighborhoods of Requena del Tapiche are needed to improve the impact on the health of older adult people in extreme poverty.

## 1. Introduction

The World Health Organization (WHO) estimates that by 2050, the population over 60 years of age will have increased by close to 2 billion worldwide, of which 20% will suffer from a mental pathology [1]. The National University of Central Peru (UNCP) published in 2015 that most older adults in the Amazon suffer from neglect, 87.5% of adults do not receive economic help for their basic needs, 100% present health problems, and 92.5% have incomes of less than 155 soles (approximately 38 euros) [2]. In addition, most do not have close relatives to be able to share their emotions and affective needs [3]. Studies conducted in Peru in 2015 [4,5] indicated that 16.2% of older adults suffer cognitive impairment, and of these, 70% are considered extremely poor, so there seems to be a relationship between emotional, economic, psychosocial deprivation and physical disabilities as an aggravating factor in the development of depression in older people. Depression is one of the most prevalent disorders worldwide, contributing to physical discomfort and decreased functional capacity and quality of life of people [6]. It is a multifactorial disease that can contribute to older adults being more vulnerable by affecting functional capacity. It is important to perform psychosocial assessments, and more so in settings with hardly any resources of trained health professionals.

According to the WHO, a person is considered old when he or she reaches 60 years of age [7]. Aging implies deterioration at the molecular and cellular level, contributing to a decline in physical and cognitive functions, a situation that leads to increased morbidity and mortality [1]. The continuous progress of improvements in society in recent years has led to an increase in the quality of life of the population worldwide, which is related to the fact that life expectancy is increasingly higher, thus changing the consideration of older adults as a stage full of satisfaction like any other, being an insufficient criterion to evaluate and define the sick older people [1,8]. In 2020, the “Mutua de Propietarios” Foundation, in collaboration with the University of Barcelona (Spain), conducted a survey of people over 75 years of age, revealing their concerns about old age: lack of mobility, loss of health, and lack of economic resources [9].

To understand the quality of life of older adults in Latin America, it is necessary to refer to the economy. According to Oliveri [10], among the 45 million older adults residing in Latin America, about 30.3% live in vulnerable situations; 14.7% live with their relatives, and 64% are the heads of household, being responsible for the largest economic contribution. Data provided by INEI in Peru reveal that only 18.4% of those over 65 years of age receive the Non-Contributory Pension (PNC) [11]. The life situation in which older people find themselves puts pressure on them to continue participating in the labor market until very old age, regardless of their physical ailments; 56.1% of the population over 60 years of age belongs to the Economically Active Population with an average of 37.5 h, of work per week, and in many other countries, it exceeds 40 h to access an adequate level of subsistence, credit markets, health insurance among other needs.

Healthcare is a good that should be fundamental for everyone in the world. However, in countries such as Peru, 20% of the population cannot access basic healthcare [12]. Of a total of 6074 registered medical specialists in the country, 70% are in the city of Lima, thus causing a deficit gap in Amazonian regions such as Loreto, the department where the city of Requena is located [13].

Depression, one of the most prevalent disorders worldwide, significantly contributes to physical discomfort and the decline in functional capacity and quality of life among individuals [14]. It is a multifactorial disease that can increase the vulnerability of older adults by affecting their functional capacity. The symptomatology differs between older people and the young, and it is common for older persons to exhibit not only sadness, social isolation, and fatigue but also memory loss, agitation, and sensory disturbances such as persecutory delusions, unexplained pain, pain inhibition, Cotard’s syndrome, among others [15,16]. However, these symptoms are often misinterpreted as initial stages of neurological and physical diseases typical of older persons. Epidemiological studies indicate that 25% of older persons suffer from Alzheimer’s disease, 33% from Parkinson’s disease, and 50% of hospitalized older persons also experience such symptoms [17].

In recent decades, life expectancy has increased thanks to advances in medicine, technology, and economics, thus favoring longevity. It is estimated that in the future, life expectancy will exceed 80 years in developed countries [18,19]. However, what appears to be an indicator of the quality of life in the country leads to governments having to face situations related to aging, such as chronic diseases and functional disabilities, that must be addressed through the allocation of health, economic, and social resources. While disability cannot be considered a direct cause of aging, the association between increasing age, frailty in older persons, and loss of autonomy is undeniable [19,20]. This dependency falls on the caregiver, usually, a close family member, who takes on the responsibility of direct care throughout most of the day, abandoning social activities and neglecting themselves, thus becoming more vulnerable to illness and the development of caregiver burnout syndrome [20,21,22,23].

In 1977, the American psychiatrist Engel proposed a modification in the biomedical model, considered until then by a new perception of the person from a holistic point of view, thus considering the biological, psychic, and social aspects as key paradigms through which diseases are somatized and reflected [24]. Even though older people may live more years with respect to the past, there are factors beyond the biological ones that harm the adult related to the passing of the years. Rondón and collaborators [25] state that there is a bidirectional relationship between affective and social deprivation and the appearance of mental disorders such as depression and anxiety, as well as a positive relationship between adherence to treatment in people with greater social support. Over the years, the concept of aging has been linked to the loss of cognitive abilities, autonomy, and chronic diseases, which stigmatize the concept of “older person” [26]. The IV Survey on Social Inclusion and Exclusion of Older People conducted in Chile left evidence of the distribution of insufficient resources and the scarce social integration of older people, a situation very similar to Peru. This problem is considered a major political challenge for the coming years worldwide [27].

Our approach is supported by family systems described by authors such as Murray Bowen [28] and depression and vulnerability models by Aaron Beck [29]. The former examines interactions within the family and how they can affect mental health and cognitive functioning. Family dynamics, communication, and support can have a significant impact on the experience of depression and cognitive decline. The second model proposes that the combination of individual vulnerability factors and environmental stressors can lead to the development of conditions such as depression. The socio-family situation could be an additional stressor.

**Figure 1:**
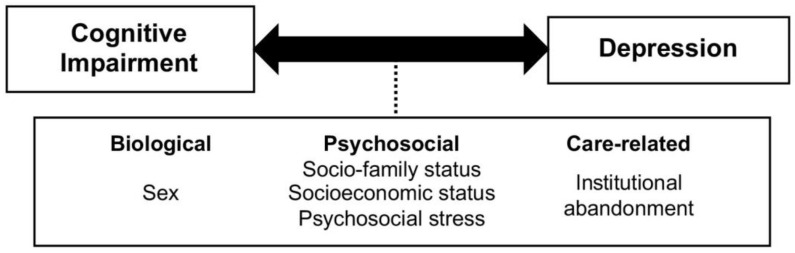
Bidirectional relationship between cognitive impairment and depression, and possible factors that regulate it.

Often, both sex and psychosocial and caregiving factors are implicated in both depression and cognitive impairment and may precipitate the impairment-depression relationship, promoting the transition between them. Figure 1 shows some of the possible mechanisms underlying this bidirectional association.

Older persons in Requena del Tapiche live in a situation of extreme poverty. The socioeconomic status favors that they live in housing that is far from dignified, and in addition, many live in a situation of loneliness, abandoned out of necessity by their family members. There are institutions of the Catholic Church that carry out silent but effective work in the most disadvantaged communities of the Loreto region, providing health resources, paying for health campaigns, and delivering basic food and household and personal belongings to improve the living conditions of older persons [30].

The Alzheimer’s Association defines mild cognitive impairment (MCI) as an early stage of memory loss or other loss of cognitive ability in people who maintain the ability to independently perform most activities of daily living [31]. Sociodemographic variables, along with cognitive abilities, undergo changes with increasing age and, with it, the loss of autonomy, with the highest peak in Moderate Cognitive Dysfunction (MCI) between the ages of 80 and 89 years [32]. Economic status is related to the quality of food received, the degree to which older adult has been involved in society, and whether he/she received an academic education or has had a skilled job, as these are aspects that can be considered as buffers against the progression of early dementia [33]. In Peru, 38.4% of the population over 70 years of age lives alone, and three out of ten elderlies go to school; there is a gap between the absence of educational matters and the population living alone [34].

For this study, information on the living conditions of the participants was collected through direct observation of their homes, taking advantage of international cooperation health visits. During these visits, sociodemographic data and data on psychosocial variables were also collected through questionnaires. Our study aims to primarily analyze family function, social risk, as well as the degree of depression and cognitive impairment in this particular population. We aspire to identify patterns and relationships that may contribute to the understanding of factors impacting the mental health and well-being of older adults in situations of extreme economic vulnerability.

## 2. Materials and Methods

The present study was carried out in the town of Requena del Tapiche, a municipality in the Loreto region of Peru, about 852 km from Lima (the capital of Peru). It is surrounded by the Tapiche River, which flows into the Ucayali River, one of the rivers into which the Amazon River forks. The way to get around is by boat, and the soil is not arable. Most of the population descends in a high percentage of aborigines such as the Cocamas, Mayorunas, Mayos, Matsés, Shipivo, and Remos. There is a tendency for native communities to migrate to Requena to improve their living conditions. In the 2012–2020 period, life expectancy at birth in the Department of Loreto was 70.1 years for men and 74 years for women. The population density is around 2 inhabitants/km^2^. In Requena, there is a range of monetary poverty rate of about 70% and a range close to 80% with more than one important basic need. Approximately half of the population is engaged in agriculture, and 60% do not have any health insurance [8,11].

This is an observational, descriptive, and cross-sectional study including all older people who attended the Requena del Tapiche Health Center (Peru) in the year 2022, excluding people who did not reside in Requena and the peripheral towns. The census of people over 60 years of age in Requena was 2233 people [35], of which 90 was the total population of older people who attended this health center, representing a proportion of 4.03%. Using the usual traditional formula for estimating a proportion and taking this data as the expected proportion, for a confidence level of 95% and margin of error of 5%, the result yields a value of 58 persons; therefore, we estimate the obtained sample size of 60 to be sufficient. Thus, not only was the sample size calculated in the traditional way, but also the effect size was calculated using G*Power software v.3.1.9.6 [36]. Thus, for between-participant comparisons, a minimum size of 60 participants is suggested for a large effect in non-parametric comparisons or a suggested minimum sample size of 50 if a parametric approach is chosen. For a bivariate model, the suggested minimum sample size is 19 participants.

### 2.1. Data Collection Procedure

For data collection, all older people who met the sample selection criteria were listed by population areas of Requena and surrounding areas. The Health Center was notified of the visits by means of posters and loudspeakers. It was preferred to collect data at the patient’s home to directly observe their living conditions, planning home visits for three weeks. The team of professionals was composed of Peruvian and Spanish nurses. All the necessary material was transported from Spain (data collection booklet with the selected scales, informed consent, and participant information sheets).

The data collection period was in the months of June and July 2022. The main purpose of the visits was to assess each older person and, in turn, to provide personalized health care. The visits were used to provide basic foodstuffs and medicines obtained through donations for international cooperation. Notes on the living conditions to be able to carry out this work were obtained through direct observation, taking notes once they consented to participate. The questionnaires were filled out by asking them directly since most of them were illiterate. The measurement scales are described below:

### 2.2. Family Apgar

This is a scale that provides graphic and schematic information about the family function, how it is related, and the global perception that the person has about the functioning of his or her family at a specific moment [37,38,39]. Its interpretation is as follows: Normofunctional (score 7–10), Mild dysfunction (score 4–6), and Severe dysfunction (0–3). It has an intraclass correlation coefficient of 0.86, an item-scale correlation between 0.61 and 0.71, and a Cronbach’s alpha value of 0.84.

### 2.3. Yesavage Geriatric Depression Scale

Depression in older persons presents several differential characteristics with respect to other age groups since older persons are more vulnerable to present depressive symptomatology due to psychosocial factors (economic difficulty, social isolation, loss of loved ones…), presence of cognitive deficits, biological changes due to aging that produce coexistence of several medical problems and increased use of medications [40,41]. This scale is designed for people over 65 years of age; the questionnaire consists of 15 dichotomous questions [42,43]. Its interpretation is as follows: Normal (score 0–5), Moderate depression (6–10), and Severe depression (greater than 10). Its sensitivity is 86%, and its reliability is 0.95.

### 2.4. GIJON Socio-Family Assessment Scale

It is a scale that assesses social-family risk with five items: family situation, economic situation, housing conditions, social relationships, and social network supports [44,45]. Its interpretation is as follows: Good social situation (5–9), social risk (10–15), and social problem (>15). It has an intraclass correlation coefficient (interobserver reliability) of 0.957 and a Cronbach’s alpha coefficient of 0.447.

### 2.5. Pfeiffer Questionnaire

It is a brief questionnaire that assesses orientation, memory, abstraction, calculation, and attention [46,47]. This questionnaire yields a numerical number from 0 to 10 whose interpretation is: No impairment (score 0–2), Mild impairment (score 3–4), Moderate impairment (score 5–7), and Severe impairment (score 8–10). It has a sensitivity of 68%, a specificity of 96%, a test-retest reliability of 0.82, and a validity of 0.76.

### 2.6. Statistical Analysis

Quantitative variables are presented using the mean and its standard deviation (SD). Qualitative variables are expressed as values and percentages (%). Normality was tested using the Shapiro–Wilk test. Normality was not found, so Spearman’s non-parametric correlation test was used to determine the degree of correlation of the variables “family function”, “social risk”, “depressive symptoms”, and “cognitive impairment”. Differences between quantitative variables were analyzed using the Mann–Whitney U test. A risk analysis was used between the sex categories and the four categorical variables mentioned above, presenting the results as Odds Ratio and its 95% confidence interval. As there were no missing data, no multiple imputation process was necessary. A significance level of less than 0.05 was accepted for all tests. SPSS v.23 software (SPSS Inc., Chicago, IL, USA) was used for the analyses.

### 2.7. Ethical Aspects

The principles of the Declaration of Helsinki [48] were complied with. Informed consent was signed by older persons. The UCV/2021-2022/202 research study obtained a favorable report from the Ethics Committee of the Catholic University of Valencia.

## 3. Results

The study was carried out in the Peruvian Amazon in a sample of 60 older people, whose ages ranged from 60 to 100 years, with a mean age of 79.2 (SD ± 6.67). The highest percentage of older people were between 70 and 80 years of age. Thirty-three women and 27 men participated, representing 55% and 45%, respectively. Table 1 describes the sociodemographic characteristics of the sample.

Requena is distributed in districts; the urban core is the commercial area, where greater purchasing power is observed. Older persons participants have their homes in the periphery. The people under study were visited and interviewed, being able to directly observe their living conditions. The houses are made of wood extracted from the jungle, and the roofs are made of a plastic-like material known as calamine, which protects them from rain and wind. Some participants’ houses have plastic walls.

Basic physiological needs are not covered among people living in extreme poverty, a situation that becomes more complicated as the years go by. We found that 55% of the study population drinks untreated well water. Only 37% make their water drinkable with sodium hypochlorite to avoid parasitosis.

Half of older persons do not have a latrine to meet their disposal needs, and if they do, it is located away from the house. They are usually shared among neighbors and consist of a wooden hut with a hole in the floor for depositing excrement, with a water drum on the outside. The wood is usually in poor condition due to humidity, and it is common to find worms, flies, and mosquitoes. Access is usually dangerous because the terrain has slopes and descents full of rocks, leaves, and logs, aggravated by mud during the rainy season.

Most people live in one-room houses where all family members cook, live, and sleep together. Many rest on the ground or on boards that protect them from the humidity. 

We did not find statistically significant differences in age or sex depending on family functional status. It is noteworthy that 50% indicated that they never have a relationship with their neighbors, finding themselves helpless in the event of an emergency (Table 2).

According to the results of the GIJON scale, the social situation of older persons is good in only one case, which corresponds to a woman. Ninety-nine percent are at risk of social exclusion or with a real social problem already established. In 38% of the cases, the family is formed by themselves because they live alone, the children live far away, and the partner separated or died a long time ago. More than half of them live with a relative, which makes them feel more accompanied. Sixty-three percent of older persons reported having a poor economic situation, receiving no income at all or only a minimal income every two months (Table 3).

More than 60% suffer from some symptoms of depression, which is more frequent in women. However, they all report feeling satisfied with life despite the precarious situation that accompanies them (Table 4).

Table 5 shows the analysis of the characteristics of the older adults according to the results of the Pfeiffer scale of cognitive impairment.

Table 6 shows the analysis of correlations between the different variables representing family function, social risk, degree of depression, and cognitive impairment. In this analysis, we found an inverse correlation between family functioning and cognitive impairment (r = −0.234; *p* = 0.046). Also, an inverse correlation between family functionality and social risk (r = 0.514; *p* = 0.000) and, finally, a direct correlation between the risk of depression and social risk (r = 0.526; *p* = 0.023). All the above indicates that there is an interrelation between family functionality and social and depression risk. The lower the family functionality, the higher the social and depression risk in older persons.

Cognitive deterioration is an aspect to be considered as the years go by, which can be increased by nutritional deficits, social exclusion, poverty, and lack of autonomy, among others. Loneliness in older people can have a serious impact on cognitive decline as the years go by. Nearly 70% of people who have been found to be cognitively impaired live unaccompanied and in extreme poverty. Very often, people interviewed with difficulty remembering their date of birth or their address, among other things, showed signs of sadness and a considerable score on the Yesavage test. We observed that more than half of the people with cognitive impairment and depression live alone.

## 4. Discussion

The present study was carried out in the area with the largest indigenous population in the country [49], in Requena del Tapiche in Peru. According to Varela Pinedo [50], the quality of life of older people is influenced by the economy, culture, and their relationship with society, as well as by physical factors that affect their health or health policies that provide accessibility to medical treatment. In the region under study, in 2017, monetary poverty was found to be between 33.3% and 36.8%, remaining above the national average [51]. About 37% of older persons receive the minimum wage, 40% have a minimum non-contributory pension of 300 soles (about €74) every two months, and the remaining 23% do not receive any type of financial assistance [52,53].

In rural areas of Peru, it is difficult to determine the age of retirement, as older persons continue to work into old age [10,11] to make ends meet. The economic problem is because only one-third of the Peruvian population has a job that requires affiliation to a pension and health system [54]. When they reach old age, these people do not have sufficient resources to pay for food and basic medical care. Older persons do not go to health centers when they fall ill [51] because 10–20% of the population is totally excluded from the health system [54].

Rest is one of the most important needs of a person. The detection of insomnia in older persons is a challenge since it often goes unnoticed despite being considered a disorder with a great impact on mental health and cardiovascular, neurological, and psychiatric diseases [55]. To consider a good rest, it is essential to have an adequate place for it; an inadequate temperature will favor a poorly restorative rest. We observed that the participants do not have an adequate place to rest, sleeping directly on a wooden structure without a mattress or on the ground itself (which is sometimes damp earth) and in the company of animals, which leads to osteoarticular pain and health risks. The most privileged have a 20 cm thick mattress provided by an institution of the Catholic Church (Caritas).

It is essential to feel accompanied because people feel the need to love and be loved, and even more so in older adults when, in most cases, loneliness is not chosen. This sad feeling has a negative influence on mental health, causing anxiety and depression [56,57,58]. Prieto-Flores characterizes depression as a factor related to suffering loneliness. Therefore, we would be facing negative feedback that has an impact on the health of older persons, causing, in many cases, symptoms that could be confused with cognitive impairment [59]. As has been observed, a large part of the population under study lives alone in conditions of extreme poverty, where moving to the water well or latrine is a difficult task due to the slopes and slippery terrain, which favors falls and their consequences, such as physical and psychological injuries.

Affective isolation is defined as a state of objective loneliness in which the person feels socially excluded and helpless from others [60]. The authors Gale and Gerino, with their collaborators [61,62], associate loneliness with the risk of suffering depression, physical immobility, and anxiety. A large proportion of older persons suffer from depressive symptoms, coinciding with the fact that they live alone.

We agree with the results of Molés and collaborators [63] in the fact that it is women who presented a greater proportion of depressive symptoms. However, we do not agree with Sánchez García and colleagues [64], who state that the frequency of depression is higher in people aged 85 years and older. Analyzing the figures in our study, we obtained a higher degree of loneliness in those aged between 70 and 90 years; therefore, in our sample, depressive symptoms appear in a younger population.

Zhong et al. [65] found a relationship between loneliness, depression, and cognitive impairment, a reality that, in turn, is indirectly implicating the person’s loneliness with the dementia that may develop. Memory loss is one of the most common symptoms in people with diagnosed depression [66]. Bennett concludes that in patients with previous dementia, cognitive impairment increases if, in addition, they are diagnosed with depression [67].

In our study, we have found an interrelation between family functioning and social and depression risk. As it seems logical, the lower the family functionality, the higher the social and depression risk in older people. Likewise, we have detected many cases of cognitive impairment. Validated standardized scales and assessment instruments are tools that help nurses work with great scientific rigor [46]. We observed that many of those with cognitive impairment and established depression live alone. Loneliness is a risk factor. Different studies [68,69,70] recognize loneliness as a social and public health problem; physical-affective deprivation in older people predicts continued hospital admission. Kadowaki and collaborators [71] conclude that those older adults who have home social assistance have lower levels of loneliness and stress and higher levels of life satisfaction. To remedy this unchosen loneliness in older persons, it is necessary for Primary Health Care to establish community activities in which people can interact and thus establish supportive and close social relationships. It is worth highlighting the important work of the nurse at the Requena Apostolic Vicariate Health Center, who carries out health campaigns, distributes basic foodstuffs, and provides health promotion interventions to teach good lifestyle habits and, in addition, provide support to people who live in absolute loneliness [28].

According to the data collected by means of the GIJON scale [44,45], we observe that practically the totality of the population under study presents an established social problem or is at social risk; only one woman has a normal social situation. Caring for older persons is a family responsibility, specifically for the children. However, due to sociodemographic changes, the young population is increasingly migrating to large cities in search of better conditions in education, health, and economy, which is a problem for the older population who remain in rural areas without family support [72].

Even though many older people state that they have a normal family support situation, they also indicated to us that they do not feel satisfied with the support received by their families when they have a problem. They are apparently happy. During the home visits, by offering humanized, close nursing care in a climate of intimacy and trust, they expressed to us their real feelings of loneliness. The highest scores on the family Apgar scale were obtained at older ages, as this coincides with those older people who live with their children, who are usually isolated cases.

In Peru, there is financial aid from the Ministry of Development and Social Inclusion for people over 65 years of age living in extreme poverty, which consists of an income every two months of 250 soles, which is clearly insufficient due on the one hand to the rise in prices since the COVID-19 pandemic and on the other hand to the fact that most of them do not receive any other pension. In 2023, this figure has been increased to 300 soles, which is equivalent to about 79 dollars or 74 euros every two months [73]. On the other hand, this aid does not reach undocumented people or those who are illiterate (of which there are many) because they are unable to properly complete the application documents [74].

Peru has a network for the protection of older persons with disabilities, but in this part of the Amazon, the assistance does not reach and is not known, even though it is being developed in 152 municipalities in the country [75]. Public initiatives continue to be scarce and inefficient. Institutions such as Caritas (of the Catholic Church), Universities, and NGOs are longitudinally carrying out interventions such as monthly food deliveries, nursing, and medical care in their charitable health centers.

The situation in which older persons live reflects the extreme poverty to which they are subjected. From the submerged working life, migration of children, and unhealthy home conditions, older people are impaired in the quality of life both physically and psychologically [76].

The main limitations of the present work are that it is a cross-sectional study, with the biases that this type of study can cause, and the difficulty in data collection because although the research team shared the language, there are cultural barriers that caused difficulty in understanding on some occasions. On the other hand, the strength of our work lies in direct observation through home visits by the research team.

## 5. Conclusions

Older persons reside in peripheral districts of Requena in wooden houses covered by corrugated calamine roofs. They sleep on boards or directly on the ground. Latrines are shared and far from the home. The water they drink is not safe and is sometimes treated with bleach to prevent parasite infections. The family function of the rural population in the Amazon is unstructured until the age of 90. In the later stages of life, family support increases. Our analysis shows an interrelation between family functioning and social and depression risk. The lower the family functioning, the higher the social and depressive risk in older persons associated with loneliness and lack of economic resources. A large proportion of older persons interviewed presented depressive symptoms.

All this suggests the urgent need to develop more cooperative and longitudinal research projects to evaluate the impact on the health of older persons after the health campaigns and health promotion interventions developed in the suburbs of Requena del Tapiche.

## Figures and Tables

**Table 1:** Sociodemographic characteristics of the sample.

	*n* (%)
Sex	
Male	27 (45.0)
Female	33 (55.0)
Age ranges	
60–70 years old	6 (10.0)
70–80 years old	29 (48.3)
80–90 years old	22 (36.7)
>90 years old	3 (5.0)
Housing construction material	
Wood	50 (83.3)
Brick	7 (11.7)
Tambo	2 (3.3)
Plastic	1 (1.7)
Roofing material	
Calamine	55 (91.7)
Leaves	5 (8.3)
Drinking water	
Bottled	11 (18.3)
Treated	22 (36.6)
Well water	27 (45.0)
Latrines	
Sanitary	30 (50.0)
Non-sanitary	22 (36.6)
No toilet	8 (13.3)
Resting conditions	
Mattress	26 (43.3)
Floor	8 (13.3)
Table	26 (43.3)

**Table 2:** Characteristics of older adults according to family functionality status (Family APGAR Scale).

Family Functionality				
Parameter	Functional (*n* = 36)	Dysfunctional (*n* = 24)	OR (CI95%)	*p*-Value
Age (years)	78.97 ± 7.05	79.50 ± 6.37	NA	0.779 *
Sex				
Male	15 (41.7%)	12 (50.0%)	0.71 (0.25–2.02)	0.525
Female	21 (58.3%)	12 (50.0%)		

Values are expressed as mean ± standard deviation for quantitative variables. Qualitative variables are expressed as *n* (%). *: Mann–Whitney Test; OR: Odds Ratio; CI95%: Confidence Interval 95%; NA: Not applicable.

**Table 3:** Characteristics of older adults according to their social situation (Gijón socio-family scale).

Social Situation				
Parameter	Without Social Problems (*n* = 30)	With Social Problems (*n* = 30)	OR (CI95%)	*p*-Value
Age (years)	79.23 ± 7.30	79.17 ± 6.10	NA	0.970 *
Sex				
Male	15 (55.6%)	12 (44.4%)	1.50 (0.54–4.17)	0.436
Female	15 (45.4%)	18 (54.5%)		

Values are expressed as mean ± standard deviation for quantitative variables. Qualitative variables are expressed as *n* (%). *: Mann–Whitney Test; OR: Odds Ratio; CI95%: Confidence Interval 95%; NA: Not applicable.

**Table 4:** Characteristics of older adults according to depressive symptoms (Yesavage Scale).

Depressive Symptoms				
Parameter	With Symptoms (n = 38)	Without Symptoms (*n* = 22)	OR (CI95%)	*p*-Value
Age (years)	78.37 ± 7.01	80.64 ± 5.92	NA	0.207 *
Sex				
Male	17 (63.0%)	10 (37.0%)	1.03 (0.36–2.96)	0.957
Female	21 (63.6%)	12 (36.4%)		

Values are expressed as mean ± standard deviation for quantitative variables. Qualitative variables are expressed as *n* (%). *: Mann–Whitney Test; OR: Odds Ratio; CI95%: Confidence Interval 95%; NA: Not applicable.

**Table 5:** Characteristics of older adults according to cognitive impairment (Pfeiffer scale).

Cognitive Impairment				
Parameter	With Cognitive Impairment (*n* = 41)	Without Cognitive Impairment (*n* = 19)	OR (CI95%)	*p*-Value
Age (years)	78.46 ± 6.33	80.79 ± 7.27	NA	0.212 *
Sex				
Male	20 (48.8%)	7 (36.8%)	0.61 (0.20–1.87)	0.387
Female	21 (51.2%)	12 (63.2%)		

Values are expressed as mean ± standard deviation for quantitative variables. Qualitative variables are expressed as *n* (%). *: Mann–Whitney Test; OR: Odds Ratio; CI95%: Confidence Interval 95%; NA: Not applicable.

**Table 6:** Correlations between the different variables representing family function, social risk, degree of depression, and cognitive impairment.

		Yesavage Score	Pfeiffer Score	Gijon Score	Family Apgar Score
Yesavage score	Spearman’s rho		−0.135	0.526 *	0.110
	*p*-value		0.305	0.023	0.402
Pfeiffer score	Spearman’s rho	−0.135		0.175	−0.234 *
	*p*-value	0.305		0.182	0.046
Gijon score	Spearman’s rho	0.526 *	0.175		−0.514 **
	*p*-value	0.023	0.182		0.000
Family Apgar score	Spearman’s rho	0.110	−0.234 *	−0.514 **	
	*p*-value	0.402	0.046	0.000	

*: Correlation is significant at the 0.05 level. **: Correlation is significant at the 0.01 level.

## Data Availability

Data are available upon reasonable request.
